# Whole-brain dopamine transporter binding pattern predicts survival in multiple system atrophy

**DOI:** 10.1186/s40035-024-00411-2

**Published:** 2024-04-02

**Authors:** Yeon-koo Kang, Jung Hwan Shin, Hongyoon Choi, Han-Joon Kim, Gi Jeong Cheon, Beomseok Jeon

**Affiliations:** 1 Department of Nuclear Medicine, Seoul National University Hospital, 101, Daehak-Ro, Jongno-Gu, Seoul, 03080 Republic of Korea; 2 Department of Nuclear Medicine, Seoul National University College of Medicine, Seoul, Republic of Korea; 3 Department of Neurology, Seoul National University Hospital, 101, Daehak-Ro, Jongno-Gu, Seoul, 03080 Republic of Korea; 4 Department of Neurology, Seoul National University College of Medicine, Seoul, Republic of Korea; 5 Department of Molecular Medicine and Biopharmaceutical Sciences, Graduate School of Convergence Science and Technology, Seoul National University, Seoul, Republic of Korea; 6 Institute on Aging, Seoul National University, Seoul, Republic of Korea; 7 Cancer Research Institute, Seoul National University, Seoul, Republic of Korea; 8 Institute of Radiation Medicine, Seoul National University College of Medicine, Seoul, Republic of Korea

Multiple system atrophy (MSA) is an atypical parkinsonian syndrome characterized by multi-system involvement with rapid progression and variable presentations [1, 2]. The clinical variability suggests potential subgroups with differing outcomes, emphasizing the need to identify an objective biomarker that can classify disease subgroups for disease management and clinical trials. While factors like age, sex, early autonomic symptoms, and absence of levodopa responses are associated with survival, an objective biomarker reflecting a brain-wide neurodegeneration pattern that could predict the clinical outcome of MSA has not been elucidated.

Dopamine transporter (DAT) imaging using [^18^F]fluoro-propyl-carbomethoxyiodophenyl-tropane (FP-CIT) is used to assist in diagnosing parkinsonism including MSA [3]. Although it primarily focuses on DAT binding of the striatum, FP-CIT also binds to the extra-striatal areas including the dorsal pontine area due to its affinity to serotonin transporters. Therefore, it could also reflect degeneration of the raphe nuclei, which are responsible for autonomic dysfunction [4, 5]. Previous studies have shown the association between whole-brain FP-CIT uptake patterns and clinical features of MSA [6, 7].

In this study, we aimed to develop an imaging biomarker based on the whole-brain spatial pattern of DAT binding for the prognosis of MSA. We enrolled two separate cohorts in this study: unlabeled cohort and MSA cohort. We trained an autoencoder-based unsupervised clustering model with the unlabeled training cohort including all FP-CIT PET data acquired from Jan 2015 to June 2018 in a single institution, and then the model was tested for survival prediction in the independent cohort consisting of MSA patients. Survival information was collected as of August 2020 from the National Health Information Database in South Korea. The study design is detailed in Additional file 1: Supplementary Methods and Fig. S1.

Seven hundred and ninety-six patients were retrospectively enrolled in the training cohort, and 54 clinically probable MSA patients not enrolled in the training cohort, were included in the MSA cohort. The clinical diagnosis of the training cohort, and the demographic data of both cohorts are detailed in Tables S1 and S2. The MSA cohort included 36 parkinsonian (MSA-P) and 18 cerebellar (MSA-C) subtype patients, with average age at onset of 60.6 ± 10.2 years and average disease duration of 3.8 ± 3.4 years. At the time of data collection, 51.8% had deceased, with a median survival of 6.6 [95%-CI 4.6–9.5] years. The mean follow-up duration was 60.9 ± 37.2 (range 0.7–147.4) months for all patients and 79.4 ± 36.3 (35.5–147.4) months for survivors.

FP-CIT PET images were normalized using a binding ratio (BR), calculated using the occipital cortex as a reference region. The 796 images of the unlabeled cohort were classified into four clusters using an unsupervised data-driven approach, applying an autoencoder for feature reduction and K-means for clustering. The data distribution of clusters was visualized using t-distributed stochastic neighbor embedding (t-SNE) (Fig. 1a). Clusters 1, 2, 3 and 4 comprised 169, 210, 259 and 158 patients, respectively. The characteristics of these clusters were delineated on a t-SNE map using color scales based on the BR of the caudate nucleus or putamen (Fig. 1b, c).

**Fig. 1:**
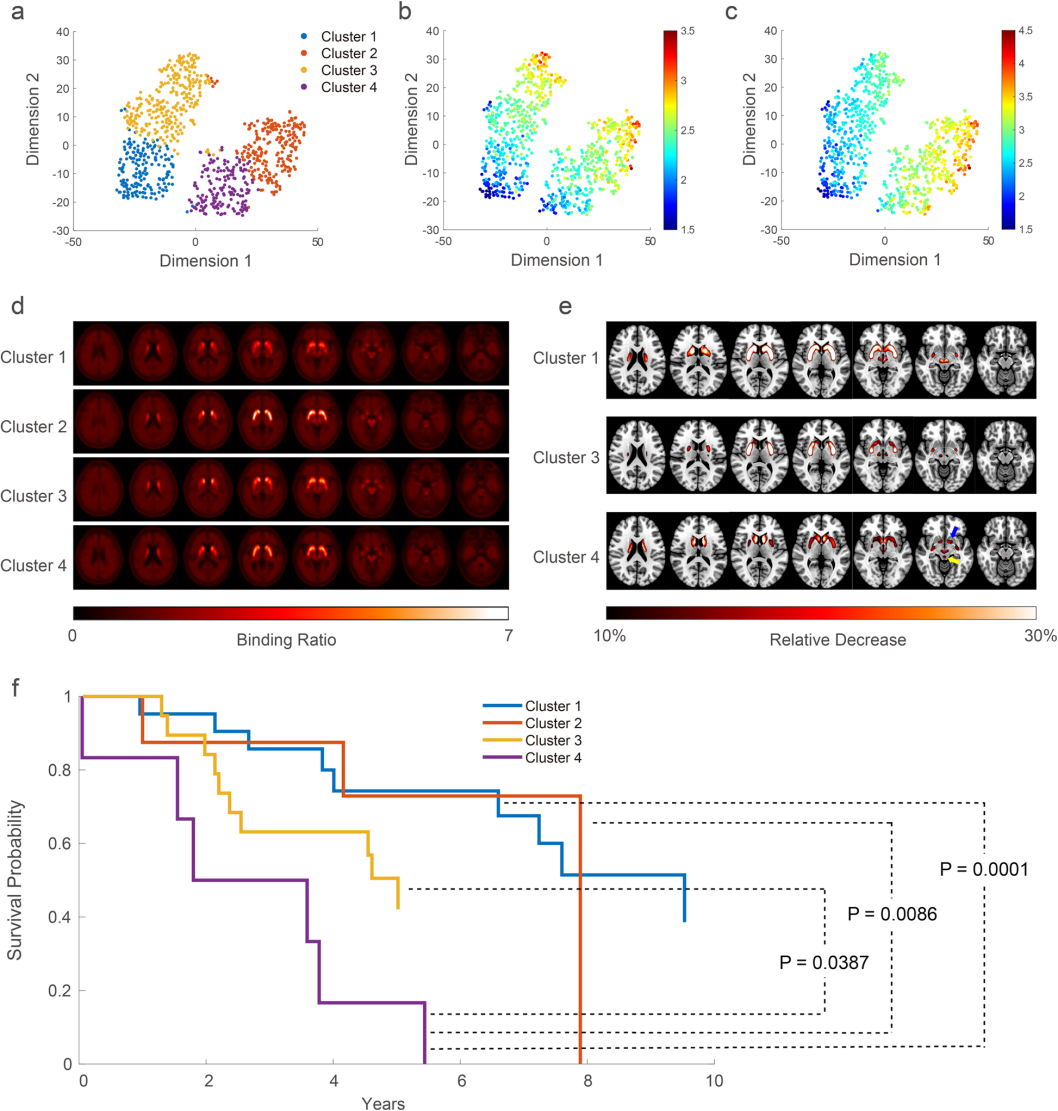
Four distinct spatial patterns of FP-CIT PET images from unsupervised clustering of the unlabeled cohort and its impact on survival in the separate MSA cohort. **a** All FP-CIT PET images in the training cohort were clustered into four clusters by an unsupervised manner. The data distribution of the clusters was visualized using t-distributed stochastic neighbor embedding (t-SNE). **b**, **c** To characterize the regional uptake pattern of the clusters, the same data were mapped based on binding ratios (BRs) of the caudate nucleus (**b**) or putamen (**c**). **d** All normalized FP-CIT PET images were averaged into a single representative image for each cluster. **e** To delineate the regional abnormality pattern of clusters, a relative decrease map was produced in each cluster using cluster 2 as a reference, and presented on an MRI template. **f** Survival outcomes of the four clusters in the separate MSA cohort were demonstrated using Kaplan–Meier curve analysis

To assess the distinct whole-brain pattern of each cluster, all the images within each cluster were averaged to create a representative single image (Fig. 1d). For quantitative analysis, we then generated "relative decrease” maps for three clusters – cluster 1, 3, and 4, using cluster 2 as a reference cluster due to its relatively intact DAT binding (Fig. 1e). The relative preservation of DAT in cluster 2 was further validated by analyzing the BRs in each striatal region (Additional file 1: Table S3). The relative decrease was defined for each voxel in each cluster as the following: Relative Decrease = (BR_2_ – BR_N_) / BR_2_, where BR_2_ and BR_N_ denote the mean BR of a specific voxel in cluster 2 and cluster N, respectively. Compared to cluster 2, cluster 1 presented diffuse decrease of FP-CIT binding in the whole striatum and the ventral midbrain. Cluster 3 exhibited decreased uptake in the putamen with anteroposterior gradient. Cluster 4 showed decreased uptake in the ventral caudate nucleus (blue arrow, Fig. 1e) and a part of the midbrain, including the raphe nucleus of the midline brainstem (yellow arrow, Fig. 1e). All findings were significant in voxel-level analysis (corrected *P* < 0.05).

The prognostic value of this clustering for survival was assessed in the separate MSA cohort. The MSA patients were distributed across all 4 clusters (Additional file 1: Table S4). Age and age of onset were significantly higher in cluster 1 compared to cluster 3 (*P* = 0.008 and 0.010, respectively). The disease duration (from disease onset to the time of PET acquisition) was not significantly different among the groups. MSA-C was dominant (7/8, 87.5%) in cluster 2 with intact DAT binding, whereas MSA-P was dominant (15/19, 78.9%) in clusters 1 and 3 with decreased putaminal binding.

Multivariate survival analysis indicated that the cluster model (HR 2.00 [1.33–3.01], *P* = 0.001) and the BR of the brainstem (HR 0.07 [0.0083–0.63], *P* = 0.018) significantly predicted survival, with age, age at onset, sex, disease duration and disease subtype as cofactors (Additional file 1: Table S5). Demographic characteristics and BR of the striatal regions did not significantly affect survival. Among the four FP-CIT PET clusters, cluster 4 which exhibited decreased binding in the caudate nucleus and raphe nucleus had the worst prognosis (*P* = 0.002 by log-rank test, Fig. 1f). The hazard ratios for clusters 1, 2, 3, and 4 were 0.54 [0.26 – 1.13], 0.68 [0.24 – 1.90], 1.15 [0.52 – 2.54], and 4.56 [0.92 – 22.73], respectively, when compared against the whole clusters. Cluster 4 exhibited a hazard ratio of 5.81 (1.10–30.69, *P* = 0.0001) compared to cluster 1, and 4.94 (1.17–20.93, *P* = 0.009) compared to cluster 2. The median survival of clusters 1, 2, 3 and 4 was 9.5 [6.6–9.5], 7.9 [4.2–N/A], 5.0 [2.4–5.0] and 1.8 [1.6–3.8] years, respectively. The clinical characteristics and PET image features of all clusters are summarized in Table S4.

In this study, we applied an unsupervised clustering method to categorize the whole-brain pattern of FP-CIT PET images in a large unlabeled cohort, and validated the prognostic value of the model in a separate cohort of MSA. We identified four distinct clusters with unique DAT binding patterns from the unlabeled cohort, which would not be derived from conventional approaches including regional image parameter analyses or supervised learning methods. The clustering model demonstrated independent prognostic predictive value, in contrast to traditional regional PET quantification parameters from the striatum, in predicting survival outcomes within the independent MSA cohort, as depicted in Table S5. Notably, MSA patients were distributed across all clusters with diverse patterns even though the training cohort did not consist of MSA patients. This heterogeneity in image patterns stands for the clinical diversity of MSA, and suggests the potential to be linked with variable clinical features.

The observations on survival outcomes across clusters suggest the importance of assessing DAT binding patterns at a whole-brain level for prognostic evaluation. Clusters 1 and 2, predominantly consisting of MSA-P and MSA-C subtypes, respectively, showed no significant survival differences, aligning with previous reports [2, 8]. Conversely, poor survival was observed in cluster 4 with a higher proportion of MSA-C, which may suggest heterogeneity within the MSA subtype in terms of prognosis. This heterogeneity was observed in the effect of regional DAT binding, where cluster 1 showed better survival despite widespread striatal DAT depletion, whereas clusters 3 and 4 with decreased binding in the putamen or caudate nucleus had worse outcomes. This pattern suggests that the prognostic influence of DAT binding in specific striatal regions does not uniformly affect survival as described in the survival analysis using the BRs. It highlights the importance of comprehensive assessment of the entire pattern of DAT degeneration in the brain for a nuanced understanding of disease pathogenesis and subtyping, which in turn correlates with prognosis.

The survival analysis incorporating whole-brain DAT binding patterns provides novel insights into the pathological process related to the prognosis of MSA. Notably, cluster 4, associated with the worst prognosis, was uniquely characterized by decreased binding in the dorsal brainstem, a region also identified as prognostically significant in regional BR analysis. Decreased DAT binding in the dorsal brainstem can be linked to the degeneration of the dorsal pontine area, including the raphe nuclei and periaqueductal gray matter, which are known for their roles in autonomic and respiratory functions [5, 9]. This association could contribute to impaired survival, as also evidenced by previous studies that identified early-onset autonomic dysfunction as a predictor of higher mortality in MSA [2]. The results are also consistent with reports of an association between decreased serotonergic transporter binding in the brainstem and disease severity in MSA [10].

This study has some limitations. First, the small MSA cohort and the uneven distribution across clusters in MSA, with the underrepresentation of cluster 4, may affect the generalizability of the findings. Second, clinical rating scales like UMSAR (Unified multiple system atrophy rating scale) were not incorporated. Their inclusion could enhance the understanding of the correlation with clinical manifestations. Third, the diagnosis was not confirmed through autopsy. However, the longitudinal follow-up (average 60.9 months) without change of diagnosis and the observed survival data aligning with results from autopsy-confirmed cases suggest high diagnostic accuracy [11]. Lastly, the retrospective nature of the study may have introduced selection bias, warranting the need for prospective large-cohort studies.

In conclusion, the whole-brain DAT pattern identified from unsupervised clustering, based on a large independent cohort, was associated with survival in MSA. The cluster demonstrating decreased binding in the dorsal brainstem was associated with higher mortality. The integrated whole-brain DAT patterns may provide novel insights into the heterogeneity in clinical progression and underlying pathological processes in MSA.

### Supplementary Information

**Additional file 1: Supplementary Methods. Figure S1.** The schematic flow of the study design. **Table S1.** Clinical diagnosis of the training cohort. **Table S2.** The demographic characteristics of the training cohort and MSA patients. **Table S3.** Binding ratios for each striatal regions in clusters. **Table S4.** Clinical and image-based characteristics of clusters. **Table S5.** Results of survival analysis using clinical and PET imaging factors.

## Data Availability

The datasets used and/or analyzed during the current study are available from the corresponding author on reasonable request.

## Abbreviations

BR: Binding ratio
DAT: Dopamine transporter
FP-CIT: [^18^F]Fluoro-propyl-carbomethoxyiodophenyl-tropane
MSA: Multiple system atrophy
