# Supplementary material for: Whole-brain dopamine transporter binding pattern predicts survival in multiple system atrophy
Source: Transl Neurodegener. 2024 Apr 2;13:18. doi: 10.1186/s40035-024-00411-2 (PMC10986063; doi:10.1186/s40035-024-00411-2)
Supplement: Supplementary file 1 — Additional file 1: Supplementary Methods. Figure S1. The schematic flow of the study design. Table S1. Clinical diagnosis of the training cohort. Table S2. The demographic characteristics of the training cohort and MSA patients. Table S3. Binding ratios for each striatal regions in clusters. Table S4. Clinical and image-based characteristics of clusters. Table S5. Results of survival analysis using clinical and PET imaging factors. [file 40035_2024_411_MOESM1_ESM.docx]

**Supplementary Information**

**Whole brain dopamine transporter binding pattern predicts survival in multiple system atrophy**

Yeon-koo Kang, MD, PhD^1,2†^, Jung Hwan Shin, MD, PhD^3,4†^, Hongyoon Choi, MD, PhD^1,2*^, Han-Joon Kim, M.D., Ph.D.^3,4*^, Gi Jeong Cheon, MD, PhD^1,2,5,6,7,8^, Beomseok Jeon, MD, PhD^3,4^

*^1^Department of Nuclear Medicine, Seoul National University Hospital, Seoul, Republic of Korea;*

*^2^Department of Nuclear Medicine, Seoul National University College of Medicine, Seoul, Republic of Korea;*

*^3^Department of Neurology, Seoul National University Hospital, Seoul, Republic of Korea;*

*^4^Department of Neurology, Seoul National University College of Medicine, Seoul, Republic of Korea;*

*^5^Department of Molecular Medicine and Biopharmaceutical Sciences, Graduate School of Convergence Science and Technology, Seoul National University, Seoul, Republic of Korea;*

*^6^Institute on Aging, Seoul National University, Seoul, Republic of Korea;*

*^7^Cancer Research Institute, Seoul National University, Republic of Korea;*

*^8^Institute of Radiation Medicine, Seoul National University College of Medicine, Seoul, Republic of Korea;*

^†^These authors contributed equally.

**Supplemenatry Methods**

*Participants*

We enrolled two separate cohorts in this study: multiple system atrophy (MSA) and unlabeled cohort. For the MSA cohort, we retrospectively reviewed the medical records of the patients diagnosed with clinically probable MSA according to the second consensus criteria [1] and underwent [^18^F]fluoro-propyl-carbomethoxyiodophenyl-tropane (FP-CIT) brain PET between Jan 2009 and Dec 2018. For clinical information, we collected age, sex, the onset of motor symptoms and disease duration (time interval between disease onset and the date of FP-CIT brain PET). We collected survival information as of August 2020 from the National Health Information Database in South Korea. For the unlabeled cohort, we retrospectively included all FP-CIT PET data acquired from Jan 2015 to June 2018 in Seoul National University Hospital that were performed to evaluate patients with parkinsonism and other neurodegenerative diseases. The PET data of the training cohort without diagnostic labeling was also previously used to develop an unsupervised learning model to discover the spatial pattern of FP-CIT binding in the brain [2]. All patients enrolled in the MSA cohort were not included in the training cohort. The design of the study was approved by the Institutional Review Board of Seoul National University Hospital (IRB No. 1907-100-1048 and 2012-097-1181). Informed consent was waived because of the retrospective nature of the research.

*PET image acquisition and preprocessing*

CT scans for attenuation correction and subsequent PET emission scans were performed 120 min after intravenous injection of FP-CIT (185 MBq) using a dedicated PET/CT scanner (Biograph true point with true V or Biograph mCT 40 or Biograph mCT 64, Siemens Healthineers, Erlangen, Germany). PET images were reconstructed using the ordered-subset expectation maximization (OSEM) algorithm (21 subsets and 6 iterations) and CT-based attenuation correction. The matrix size was 400 x 400 and a post-reconstruction Gaussian filter with a full-width at half maximum of 4 mm was applied. Reconstructed PET images were spatially normalized to a previously developed in-house FP-CIT PET template [3] with a voxel size of 2 x 2 x 2 mm, and were smoothed by an additional Gaussian filter with full-width at half maximum of 10 mm. Preprocessing steps were performed using Statistical Parametric Mapping 12 (SPM12, University College of London, London, UK).

*Binding ratio calculation for count normalization*

For PET count normalization, the binding ratios (BRs) of all voxels were calculated as BR = C_voxel_ / C_ref_, where C_voxel_ stands for the count of a specific voxel and C_ref_ denotes the count of the occipital cortex which was regarded as the reference region. The transformed BR images were used for the following analyses. The mean BR values for the putamen, caudate nucleus and brainstem were calculated. The voxels of interest (VOIs) for the occipital cortex, putamen and caudate nucleus were defined with a predefined anatomic atlas (AAL, Automated Anatomical Labeling). The VOI for the brainstem was drawn manually on the FP-CIT PET template. All the preprocessing processes were performed using free neuroimaging analysis software NiBabel 4.0.0 (https://github.com/nipy/nibabel) and computing software SciPy 1.7.3 (https://github.com/scipy/scipy) implanted on Python 3.7.3.

*Unsupervised clustering based on autoencoder*

For the first step, to classify FP-CIT PET image patterns in an unsupervised clustering manner, an autoencoder model was applied for all FP-CIT PET images from the unlabeled cohort (Figure 1, step 1). The voxels with FP-CIT binding were selected to generate a mask on the template space. The global mean FP-CIT PET image was obtained and a threshold higher than BR > 1.3 was used to generate a mask which resulted in 12,783 voxels. These voxels of each FP-CIT PET were used as an input for an autoencoder, which is an unsupervised or self-supervised learning algorithm widely used in dimensionality reduction and clustering. The model consisted of two parts, an encoder and a decoder. An encoder reduces the dimension of PET image data to produce low-dimension latent features, and a decoder reconstructs original data from the latent features. The model was trained using the training cohort data to minimize a loss function defined as binary cross entropy. The input values of 12,783 dimensional vectors were empirically divided by 10 to have a range of less than 1. An optimization algorithm ‘Adam’, with an epoch number of 100, a learning rate of 0.0002 and a batch size of 32 was applied. Keras 2.3.1 with Tensorflow 2.1.0 as the backend was used for model development.

The produced latent features from all the training cohort data were clustered using the K-means clustering algorithm [4]. The number of clusters was determined to be four, which was defined by the elbow method. The data distribution was visualized on a bi-dimensional plane by dimensionality reduction using t-distributed stochastic neighbor embedding (t-SNE).

For the second step, the FP-CIT images from the MSA cohort were processed using the trained autoencoder and K-means clustering models to be classified into the defined clusters (Figure 1, step 2). The cluster information of the MSA cohort was tested for prognostic significance. Scikit-learn 1.0.2 was implanted for the clustering algorithm.

*Statistical analysis*

The distribution of continuous variables was exhibited as average and standard deviation. Student’s t-test and chi-square test were applied to compare continuous and categorical parameters between groups, respectively. Voxel-level differences of brain PET images between groups were evaluated using SPM12 (University College of London, London, UK) with Bonferroni multiple comparison corrections. Prognostic analyses were performed using multivariate stepwise Cox proportional hazards regression analysis with age, sex, age of onset, disease duration and disease subtype as cofactors. General statistical analyses were performed using the open-source library SciPy 1.7.3 (https://github.com/scipy/scipy). Results with p-value less than 0.05 were regarded as significant.

References

1. Gilman S, Wenning GK, Low PA, Brooks DJ, Mathias CJ, Trojanowski JQ, Wood NW, Colosimo C, Dürr A, Fowler CJ, et al: **Second consensus statement on the diagnosis of multiple system atrophy.** *Neurology* 2008, **71:**670-676.

2. Suh M, Im JH, Choi H, Kim H-J, Cheon GJ, Jeon B: **Unsupervised clustering of dopamine transporter PET imaging discovers heterogeneity of parkinsonism.** *Hum Brain Mapp* 2020, **41:**4744-4752.

3. Kim Y-i, Im H-J, Paeng JC, Lee JS, Eo JS, Kim DH, Kim EE, Kang KW, Chung J-K, Lee DS: **Validation of Simple Quantification Methods for 18F-FP-CIT PET Using Automatic Delineation of Volumes of Interest Based on Statistical Probabilistic Anatomical Mapping and Isocontour Margin Setting.** *Nucl Med Mol Imaging* 2012, **46:**254-260.

4. Lloyd S: **Least squares quantization in PCM.** *IEEE transactions on information theory* 1982, **28:**129-137.


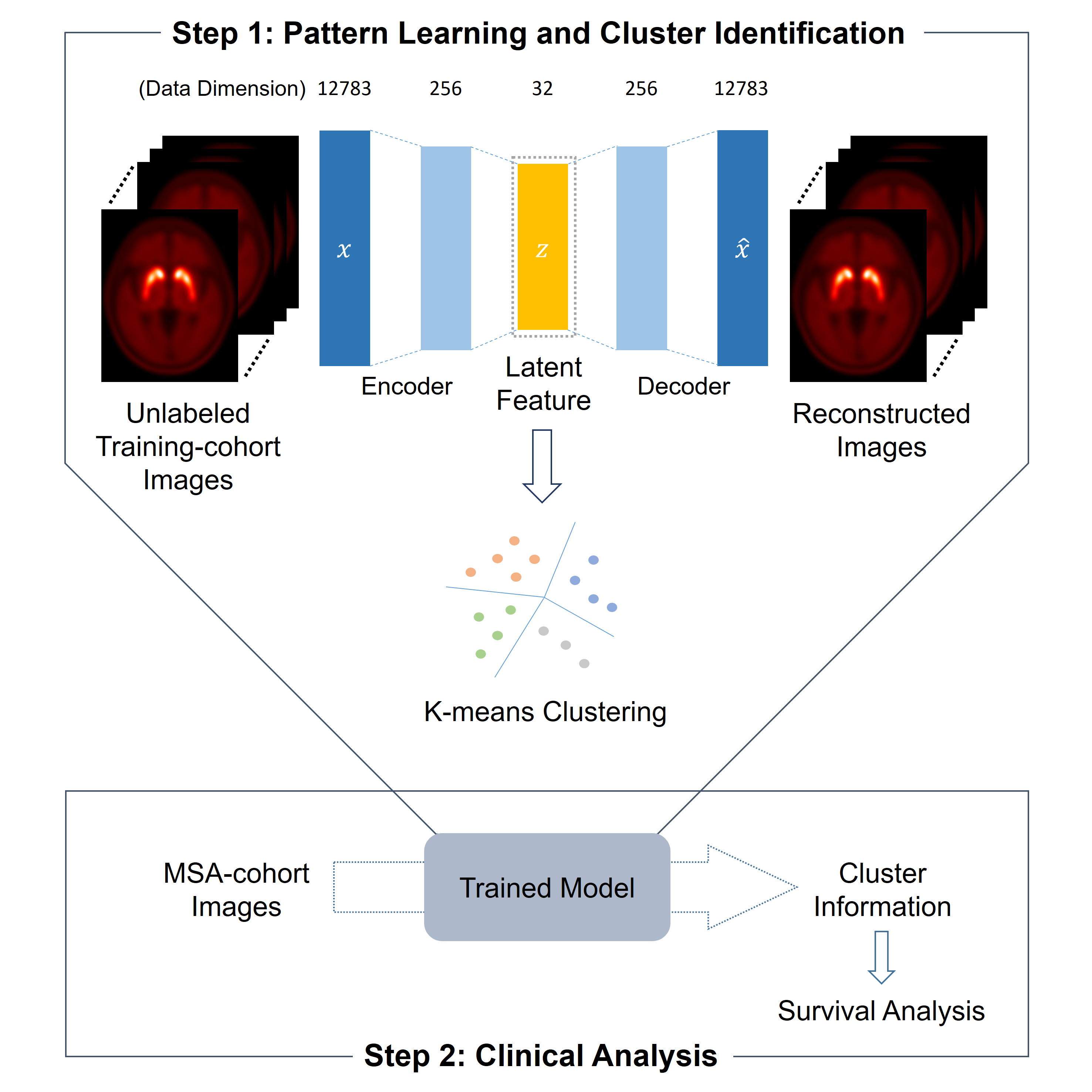


**Figure S1**. The schematic flow of the study design

**Table S1.** Clinical diagnosis of the training cohort

| Diagnosis | Number | Normal/Abnormal |
| --- | --- | --- |
| Parkinson disease | 239 | 6/233 |
| Parkinsonism, indeterminate | 186 | 31/155 |
| Parkinson plus syndrome* | 78 | 25/53 |
| Secondary parkinsonism** | 70 | 63/7 |
| Essential tremor | 111 | 110/1 |
| Gait disturbance | 44 | 44/0 |
| Dystonia | 11 | 11/0 |
| Dementia, non DLB | 24 | 20/4 |
| Functional movement disorder | 25 | 24/1 |
| Isolated cerebellar ataxia | 3 | 2/1 |
| Myoclonus | 2 | 1/1 |
| Stiff person syndrome | 1 | 1/0 |
| Fibromyalgia | 1 | 1/0 |
| Progressive bulbar palsy | 1 | 1/0 |
| Total | 796 | 340/456 |

*Progressive supranuclear palsy, Multiple system atrophy and dementia with Lewy bodies

**Drug induced parkinsonism or vascular parkinsonism

Normal/Abnormal were classified with visual interpretation of FP-CIT PET images.

**Table S2.** The demographic characteristics of the training cohort and MSA patients

|  | Training cohort | MSA cohort |
| --- | --- | --- |
| Number | 796 | 54 |
| Age | 67.6 ± 10.4 (13 – 96) | 64.0 ± 9.1 (47 – 84) |
| Sex (M:F) | 350:446 | 24:30 |
| Age at onset (year) | - | 60.6 ± 10.2 |
| Disease duration (years) | - | 3.8 ± 3.4 |
| Follow-up period (years) | - | 5.1 ± 3.1 |

**Table S3.** Binding ratios for each striatal regions in clusters

|  | Put (L) | Put (R) | Put (B) | Cau (L) | Cau (R) | Cau (B) |
| --- | --- | --- | --- | --- | --- | --- |
| Cluster 1 | 2.38 ± 0.34 | 2.28 ± 0.32 | 2.33 ± 0.31 | 2.03 ± 0.28 | 2.06 ± 0.29 | 2.05 ± 0.28 |
| Cluster 2 | 3.44 ± 0.24 | 3.30 ± 0.26 | 3.37 ± 0.25 | 2.66 ± 0.20 | 2.65 ± 0.20 | 2.65 ± 0.20 |
| Cluster 3 | 2.73 ± 0.25 | 2.63 ± 0.25 | 2.68 ± 0.23 | 2.54 ± 0.23 | 2.59 ± 0.22 | 2.56 ± 0.22 |
| Cluster 4 | 3.12 ± 0.28 | 2.94 ± 0.27 | 3.03 ± 0.27 | 2.23 ± 0.23 | 2.21 ± 0.24 | 2.22 ± 0.23 |

Put, putamen; Cau, caudate nucleus; L, left; R, right; B, both

**Table S4.** Clinical and image-based characteristics of clusters

|  | Cluster 1 | Cluster 2 | Cluster 3 | Cluster 4 |
| --- | --- | --- | --- | --- |
| n | 21 | 8 | 19 | 6 |
| Age | 67.7 ± 6.8 | 62.8 ± 10.1 | 60.5 ± 8.9 | 67.5 ± 12.0 |
| Sex (M:F) | 12:9 | 3:5 | 5:14 | 4:2 |
| Subtype (P:C) | 18:3 | 1:7 | 15:4 | 2:4 |
| Age at onset | 63.8 ± 8.9 | 60.1 ± 10.4 | 55.8 ± 9.5 | 65.2 ± 12.7 |
| Disease duration (year) | 3.9 ± 4.0 | 2.6 ± 1.3 | 4.7 ± 3.7 | 2.3 ± 2.0 |
| Follow up duration (year) | 6.2 ± 3.3 | 4.9 ± 2.2 | 4.7 ± 3.2 | 2.7 ± 1.9 |
| Median survival (year) | 9.5 (6.6 – 9.5) | 7.9 (4.2 – N/A) | 5.0 (2.4 – 5.0) | 1.8 (1.6 – 3.8) |
| Involved regions in  FP-CIT PET | Whole striatum Ventral brainstem | Intact | Posterior putamen Part of brainstem | Caudate nucleus  Part of brainstem  Raphe nucleus |

**Table S5.** Results of survival analysis using clinical and PET imaging factors

|  | Exp(b) | *P* |
| --- | --- | --- |
| Age | - | N/S |
| Sex | - | N/S |
| Age at onset | - | N/S |
| Disease duration | - | N/S |
| MSA subtype | - | N/S |
| BR_caudate_ | - | N/S |
| BR_putamen_ | - | N/S |
| BR_brainstem_ | 0.070 (0.0083 – 0.63) | 0.018 |
| Cluster | 2.00 (1.33 – 3.01) | 0.001 |

BR_caudate_, binding ratio of the caudate nucleus; BR_putamen_, binding ratio of the putamen; BR_brainstem_, binding ratio of the brainstem
